# *In vitro* inhibition of avian pathogenic *Enterococcus cecorum* isolates by probiotic *Bacillus* strains

**DOI:** 10.3382/ps/pey593

**Published:** 2019-01-18

**Authors:** Sara Medina Fernández, Marina Cretenet, Marion Bernardeau

**Affiliations:** 1 Normandie Université, UNICAEN, ABTE, 14000 Caen, France; 2 Danisco Animal Nutrition, DuPont Industrial Biosciences, Marlborough SN8 1XN, UK

**Keywords:** *Enterococcus cecorum*, *Bacillus*, probiotic, pathogen inhibition

## Abstract

*Enterococcus cecorum* is a commensal bacteria and opportunistic pathogen that can cause outbreaks of *Enterococcal spondylitis* (“kinky back”) in poultry, with a growing concern worldwide. Numerous *Bacillus*-based probiotic strains are commercially available with proven effects in supporting gut health and growth performance, but efficacy against pathogenic *E. cecorum* is unknown. This study compared the in vitro inhibitory potential of cell-free supernatants (**CFSs**) of 18 *Bacillus* strains (14 commercial probiotic strains, 1 internal negative control and 3 type strains) on the growth of 9 clinical *E. cecorum* isolates. Standardized biomass cultures of live *Bacillus* were harvested and filtered to obtain CFSs. Inhibitory potential against *E. cecorum* isolates was assessed via a microdilution assay in which the final pathogen concentration was ∼ 10^4^ CFU/mL. Absorbance (**OD**) was measured every 15 min for 15 h and used to calculate percentage growth inhibition at an OD equivalent to 0.4 in the positive control (**PC**) (pathogen but no CFS), and growth delay vs. PC. Growth kinetic responses of pathogen isolate-*Bacillus* strain combinations ranged from total pathogen inhibition to partial inhibition, lag in growth, no effect, or increased growth vs. PC. Percentage inhibition of individual isolates varied markedly among *Bacillus* strains, from 100% to −100% (growth promotion as recorded for the type strain) (*B. amyloliquefaciens* DSM7^T^). Five *B. amyloliquefaciens* CFSs produced higher average inhibition rates (>75%) than 2 out of 3 *Bacillus licheniformis* CFSs (−2.5, and −8.39% vs. PC, respectively) and 1 out of 2 *Bacillus subtilis* CFSs (7.3% vs. PC) (*P <* 0.05). Commercial strain 3AP4 exhibited the highest average percentage inhibition vs. PC (85.0% ± 7.9) and the most consistent inhibitory effect across pathogen isolates. The findings indicate that some commercially available poultry probiotic *Bacillus* strains are effective at inhibiting pathogenic *E. cecorum* in vitro, but effects are highly strain and pathogen isolate-dependent. Further work is required to confirm effects in vivo and isolate the inhibitory substances.

## INTRODUCTION

*Enterococcus cecorum* is a commensal, Gram-positive bacteria that has been identified in the intestinal tract of a diverse range of mammals and birds, including poultry (Devriese et al., 1983; Devriese et al., 1991a; Devriese et al., 1992). Non-pathogenic strains are present in the intestines of chickens from approximately 3 wk of age and apparently dominate the gut microbiota of healthy birds by 3 mo (Devries et al., 1991b). However, pathogenic strains also occur and can result in Enterococcal spondylitis (**ES**), also known as “kinky back”, a serious disease of commercial poultry production in which the bacteria translocate from the intestine to the free thoracic vertebrae and adjacent notarium or synsacrum, causing lameness, hind-limb paresis and, in 5 to 15% of cases, mortality (de Herdt et al., 2008; Martin et al., 2011; Jung and Rautenschlein, 2014). The clinical significance of *E. cecorum* infections in broilers was first described in 2002 (Devriese et al., 2002; Wood et al., 2002). Recent evidence from a variety of articles and case reports has suggested that pathogenic *E. cecorum* is emerging (or re-emerging) as a significant challenge in poultry production worldwide, causing significant losses to commercial flocks when outbreaks occur, especially in the US (Harada et al., 2012; Aitchison et al., 2014; Jung and Rautenschlein, 2014; Dolka et al., 2016; Dolka et al., 2017). The reasons for this rise are currently unclear. Proposed explanations include a general reduction in the use of antibiotic growth promoters that may create more favorable conditions for the re-emergence of pathogens and/or the emergence of clonal isolates of *E. cecorum*; recent studies have revealed evolutionary divergent genomic features and increased virulence of pathogenic strains compared with commensal strains of *E. cecorum* (Borst et al., 2015). The existence of certain predisposing factors in the bird, such as osteochondrosis dissecans lesions in the free thoracic vertebra, may also increase the pathogenicity of *E. cecorum* and likely development of ES (Borst et al., 2016). Altered prevalence of concurrent infections, changing nutritional requirements of birds or genetic selection pressures could also be at play (de Herdt et al., 2008). Against this background antibiotic alternatives to preventing and combatting pathogenic *E. cecorum* infections in poultry production are highly desirable.

Probiotics, also known as direct-fed microbials, have been produced commercially from a range of source microorganisms (bacteria, yeasts, and fungi), and have shown considerable success in poultry production in supporting gut health and improving growth performance (FAO, 2016). Many of the currently available commercial probiotics for poultry incorporate strains of *Bacillus* sp. (typically *Bacillus subtilis*, *Bacillus amyloliquefaciens*, and/or *Bacillus licheniformis*), favored because of their spore-forming capacity and associated ability to survive harsh processing conditions as well as digestion in the stomach (Cutting, 2011). Individual species and strains vary in the precise nature of their effects and their beneficial modes of action (Lee et al., 2010), but most have been selected based on their ability to reduce gut colonization by a wide range of major pathogenic bacteria including *Escherichia coli* (Wu et al., 2011; Ahmed et al., 2014; Lei et al., 2015), *Salmonella* spp. (Jeong and Kim, 2014; Park and Kim, 2014), *Clostridium perfringens* (Gebert et al., 2007; Jeong and Kim, 2014), and *Campylobacter* spp. (Fritts et al., 2000). It is biologically plausible that strains of *Bacillus* may also be effective at inhibiting pathogenic strains of *E. ceco*rum, but this has not previously been investigated in any systematic way. Pathogenic *E. cecorum* isolated from extra-intestinal sites of diseased birds are known to exhibit significant genetic heterogeneity, differ in their pathogenesis and do not always harbor known virulence genes (Borst et al., 2015; Dolka et al., 2016; Dolka et al., 2017). Therefore, it is likely that different clinically isolated strains of *E. cecorum* may differ in their pathogenicity and potentially also in their susceptibility to the inhibitory effects of probiotic *Bacillus* spp.

This study aimed to systematically evaluate the capacity of a range of commercially produced strains of probiotic *Bacillus* spp. to inhibit or delay the growth of *E. cecorum* isolates, in vitro via the synthesis of antimicrobial compounds. The *E. cecorum* strains were sourced from broilers showing clinical signs of ES and were collected from avian production sites located in two geographical markets (US and EU) across several years, in order to maximize the genetic diversity of the pathogen represented in the study.

## MATERIALS AND METHODS

### Enterococcus cecorum *Isolates and Culture Conditions*

Isolates of 9 different clinical strains of pathogenic *E. cecorum* were obtained from the internal collections of DuPont Industrial Biosciences, or purchased from Poulpharm (Izegem, Belgium). The identification of each pathogenic isolate was confirmed by PCR or MALDI-TOF mass spectrometry. All strains had originally been isolated from extra-intestinal lesions and confirmed ES outbreaks in poultry production (broilers or breeders) allowing confidence that the tested strains were virulent and capable of causing disease. Details of the isolates and their origin are given in Table 1.

**Table 1.** Origin and details of the pathogenic *E. cecorum* isolates (n = 9).

Geographic origin of isolate	Year of isolation	Strain designation	Identification confirmation	Biological origin	Supplier
North America (NC) Carolina)	2013	12147-1	PCR + 16S	Broiler, spinal abscess	DuPont Internal Collection
North America (NC) Carolina)	2013	12696M-1	PCR + 16S	Broiler, spinal abscess	DuPont Internal Collection
North America (NC) Carolina)	2013	11957-3	PCR + 16S	Broiler, spinal abscess	DuPont Internal Collection
North America (NC) Carolina)	2013	11951-1	PCR + 16S	Broiler, spinal abscess	DuPont Internal Collection
European Union (BE)	2014	E.59.56	MaldiTof	Broiler, femoral head	Poulpharm, Izegem, Belguim
European Union (BE)	2015	F.68.19	MaldiTof	Broiler, joint	Poulpharm, Izegem, Belgium
European Union (BE)	2013	C.34.19	MaldiTof	Broiler, bone marrow	Poulpharm, Izegem, Belgium
European Union (BE)	2013	D.42.11	MaldiTof	Broiler, joint	Poulpharm, Izegem, Belgium
European Union (BE)	2015	G.75.17	MaldiTof	Broiler, articulation	Poulpharm, Izegem, Belgium

NC: North Carolina

BE: Belgium

Isolates were supplied in frozen vials in culture media and glycerol and were subsequently cultured in Brain Heart Infusion broth (**BHI**, Conda, Madrid, Spain) upon arrival in the laboratory to check for viability and purity. Aliquots were frozen in vials and stored at −80°C prior to further use.

### Bacillus *Strains and Preparation of Cell Free Supernatants (CFS)*

The inhibitory potential of 14 different commercial strains of probiotic *Bacillus*, 1 internal negative control and 3 *Bacillus*-type strains were tested. These included both DuPont proprietary probiotic strains and *Bacillus* strains isolated from other poultry probiotic products available on the market in 2015. The origin and identity of the *Bacillus* strains used in the study are given in Table 2. The DuPont proprietary *Bacillus* strains were supplied in-house. All other strains were purchased and isolated in triplicate from 3 separate production batches. All strains were identified by Illumina sequencing to ensure that the strains recovered matched those declared on the product label.

**Table 2.** Origin and identity of the probiotic *Bacillus* strains used in this study.

Probiotic Product	Manufacturer	*Bacillus* species	Internal strain designation	Commercial strain designation
Enviva^®^Pro	Danisco Animal Nutrition, DuPont Industries, US	*B. amyloliquefaciens*	15AP4	PTA-6507
Enviva^®^Pro	Danisco Animal Nutrition, DuPont	*B. amyloliquefaciens*	BS8	NRRL B-50104
Enviva^®^Pro	Danisco Animal Nutrition, DuPont	*B. amyloliquefaciens*	2084	NRRL B-50013
Calsporin	Calpis Co. Ltd, Japan	*B. amyloliquefaciens*	#4–1	C-3102/DSM 15544
Clostat	Kemin Industries Inc., US	*B. amyloliquefaciens*	#1–1	unknown
Sporulin	Novus International, Inc., US	*B. amyloliquefaciens*	#10/4	unknown
Sporulin	Novus International, Inc., US	*B. amyloliquefaciens*	#10B/1	unknown
Sporulin	Novus International, Inc., US	*B. amyloliquefaciens*	#10B/4	unknown
GalliPro	CHR Hansen, Denmark	*B. subtilis*	#11/1	DSM 17299
Galliprotect	CHR Hansen, Denmark	*B. licheniformis*	#12/1	DSM 17236
CSI	DuPont	*B. amyloliquefaciens*	22CP1	n/a
CSI	DuPont	*B. amyloliquefaciens*	3AP4	n/a
CSI	DuPont	*B. amyloliquefaciens*	BS18	n/a
CSI	DuPont	*B. amyloliquefaciens*	ABP278	n/a
Internal negative control	DuPont	*B. amyloliquefaciens*	BS27	BS27
n/a	DSMZ, Germany	*B. amyloliquefaciens*	DSM7^T^	Type Strain
n/a	DSMZ, Germany	*B. licheniformis*	DSM13^T^	Type Strain
n/a	DSMZ, Germany	*B. subtilis*	DSM10^T^	Type Strain

n/a: not applicable

Cell-free supernatants (**CFS**) were prepared from *Bacillus* strains according to the following procedure: a small amount of frozen *Bacillus* from the stock was removed with a sterile inoculating loop and streaked onto tryptic soy agar (TSA, Biokar Diagnostics, Beauvais, France) plates for overnight culture at 32°C. The next day, a small amount of the respective colony was added to 10 mL tryptic soy broth (TSB, Biokar Diagnostics, Beauvais, France) in a 50 mL conical tube and shaken at 100 rpm at 32°C for 6 to 8 h. Cultures were then streaked onto TSA plates and incubated at 32°C for 24 h to check for purity. A 10 μL aliquot of the pure culture was then transferred to a 250 mL Erlenmeyer flask containing 50 mL TSB. Flasks were shaken at 100 rpm at 32°C for 16 h, re-checked for purity, and then the culture was diluted 1:10 in Luria-Bertani broth (containing tryptones 10 g/L, NaCl 10 g/L and yeast extract 5 g/L) before measuring absorbance at an optical density (OD) of 600 nm, using a microplate reader (CLARIOstar, BMG labtech). The aim was to compare CFSs obtained from bacterial growth with comparable OD fixed at 4 ± 0.5 (equivalent to 1.10^9^ CFU/mL). A blank sample of the TSB culture medium was used to calibrate the spectrophotometer. Once cultures had reached the required absorbance range, the *Bacillus* growth was re-checked for purity and transferred to a sterile 250 mL bottle and centrifuged at 10,000 rpm for 10 min. The supernatant was then transferred into a 50 mL tube, centrifuged again at 8,000 g for 5 min, and the supernatant filtered through a 0.2 μm Nalgene filter (ThermoFisher Scientific) to obtain CFS. The CFSs were stored at −20°C until further use.

### Enterococcus cecorum *Inhibition Assay*

*Enterococcus cecorum* isolates were inoculated from deep frozen stock cultures in a BHI broth and a BHI agar plate (to check purity) and incubated overnight at 37°C. All strains were subcultured at least twice before inclusion in the assay to ensure adaptation to the growth medium. The *E. cecorum* cultures were then diluted in Tryptone Salt Broth (Casein enzymic hydrolysate 1 g/L; sodium chloride 8.5 g/L) and ino-culated in BHI medium (pre-heated for 1 h at 37°C to avoid thermic shock of *E. cecorum* cells) in 96-well UV-treated microtiter plates with flat-bottomed wells, at a final concentration of 10^4^ CFU/mL. The Frozen CFS samples were thawed at room temperature for 30 min, and then administered to the 96 well-plate into the treated wells (10% v/v) containing BHI and *E. cecorum* isolates (1% v/v). Non-treated wells contained BHI and *E. cecorum* isolate (1% v/v) only. This produced the following treatments: positive control (BHI medium plus 1% (v/v) *E. cecorum* culture) (PC); negative control (BHI medium) (NC); CFS only (BHI medium plus 10% (v/v) CFS); *E. cecorum* plus CFS (BHI medium plus 1% (v/v) *E. cecorum* culture plus 10% (v/v) CFS). All microtiter plates were covered and incubated at 37°C for 15 h in a FlexStation^®^ multi-mode microplate reader coupled with Soft Max pro software (Molecular Devices LLC, US), for the measurements of optical densities (following homogenization) at 595 nm every 15 min.

The OD data (averaged across two biological replicates with SD < 0.05) were used to calculate the percentage of pathogen growth inhibition, where inhibition is defined as the percentage reduction in growth in the experimental sample compared with that in the respective PC sample (containing pathogen in BHI medium but no *Bacillus* CFS), with growth being measured as biomass (absorbance OD), and being determined at the time-point equivalent to that producing an OD of 0.4 in the PC (equivalent to 1.10^7^ CFU/mL). This OD had been selected during validation of the experimental procedure as being indicative of the middle of the exponential growth phase of the pathogen. The following equation, where “t” is time, was used to calculate the percentage of inhibition:

\begin{eqnarray*}
&&\%{\rm inhibition} = [1 - (({\rm ODa} - {\rm OD0})/\\
&&\qquad\qquad\qquad\,\,({\rm ODb} - {\rm ODc}))]*100\nonumber\\
&&{\rm where}:\\
&& {\rm ODa} = {\rm OD}\ E.\ cecorum\ {\rm plus\ CFS, \ at\ t \ equivalent}\\
&&\qquad\qquad {\rm to\ OD\ 0.4\ of\ PC}\\
&& {\rm OD0} = {\rm OD\ of}\ E.\ cecorum \ {\rm plus\ CFS\ at\ t0}\\
&& {\rm ODb} = {\rm OD\ of\ PC\ closest\ to}\ 0.4\\
&& {\rm ODc} = {\rm OD\ of\ control\ at\ t0}. 
\end{eqnarray*}

The kinetics of *E. cecorum* growth in the presence/absence of the *Bacillus* CFSs were also studied. The delay in pathogen growth in the presence of *Bacillus* CFS was calculated as the difference in time (minutes) to reach an OD of 0.4 between the PC and CFS plus *E. cecorum* supplemented wells.

### Statistical Analysis

The results of the inhibition assays were averaged across duplicates and then analyzed by analysis of variance (ANOVA) to investigate the differences among *Bacillus* strains in their inhibitory effects. Post-hoc means separation was achieved using Tukey's Honest Standard Difference test. Statistical analysis was performed using the Fit Model platform of JMP 11.0 (SAS Institute Inc., Car, NC, 1989–2013). Differences were considered significant at *P* < 0.05.

## RESULTS

A variety of different growth kinetic responses were exhibited by the *E. cecorum* isolates when exposed in vitro to the *Bacillus* CFSs. Graphical representations of these are provided in Supplementary material S1. For some *Bacillus* CFS-*E. cecorum* isolate combinations, total or almost total pathogen inhibition was evident throughout the exposure period (15 h or 900 min). For others, pathogen growth tracked that of the PC during the initial lag phase but was partially/totally inhibited during the exponential growth phase (compared with the PC). A further response was seen in which pathogen growth tracked that of the PC but was delayed by 1 to 2 h and modified by an extended lag phase, leading to a reduced final microbial population. Conversely, in a few cases the CFS-promoted pathogen growth leading to a reduced lag phase and increased final microbial population, and in yet other cases there was no effect of the CFS on pathogen growth.

**Figure 1.**
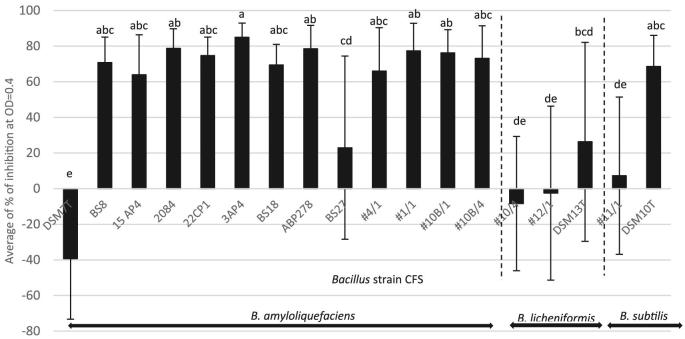
Mean percentage growth inhibition 1 of pathogenic *E. cecorum* isolates (based on 2 biological replicates; n = 9) by 18 *Bacillus* cell-free supernatants, measured at a time-point equivalent to that which produced an optical density of 0.4 in the positive control.^1^ Defined as the percentage reduction in pathogen isolate growth in the experimental sample compared with that in the respective PC sample (containing pathogen in BHI medium but no *Bacillus* CFS), with growth being measured as biomass (absorbance (OD)), not as CFU, and being determined at the time-point equivalent to that producing an OD of 0.4 in the PC. Thus, 90% inhibition would mean a 90% reduction in growth (biomass). ^a, b, c, d, e^ Bars with no common letters are significantly different (*P* < 0.01).

Figure 1 shows the mean percentage growth inhibition (biomass reduction as measured by sample absorbance) of the 9 pathogenic *E. cecorum* isolates by the 18 *Bacillus* CFSs, determined at a time-point equivalent to an OD in the PC of 0.4 (results for each pathogen isolate individually are displayed in Supplementary material S2). Except for the *B. amyloliquefaciens* type strain (DSM7^T^), all of the *Bacillus* CFSs were capable of inhibiting the growth of one or more of the pathogenic *E. cecorum* isolates, but effects varied markedly both between and within *Bacillus* species and strains (Figure 1). Percentage inhibition (vs. PC) across individual *Bacillus* strain-pathogen isolate combinations ranged from as low as −108%, indicating markedly increased pathogen growth in the presence of the CFS (as in *B. amyloliquefaciens* type strain DSM7^T^ against *E. cecorum* isolate 11951-1), to 100%, indicating total inhibition of pathogen growth (as in *B. amyloliquefaciens* CFSs BS8, 15AP4, 2084 and #10B/4 against *E. cecorum* isolate 11957-3, and *B. amyloliquefaciens* CFS #1/1 against *E. cecorum* isolate 12696M-1). Si-gnificant variation in response across *E. cecorum* isolates was also evident, to the extent that some of the *Bacillus* CFSs inhibited the growth of certain isolates of *E. cecorum* (vs. PC) whilst promoting the growth of others (Supplementary material S2). This was particularly evident in the case of CFS BS27–a DuPont internal control strain known not to exhibit strong and consistent antimicrobial potential against Gram-positive and Gram-negative bacteria (data not shown)–which showed very wide variation in inhibitory effects across pathogen strains.

The majority of the CFSs obtained from the existing proprietary *Bacillus* probiotic strains that were available on the market in 2015 belonged to *B. amyloliquefaciens*. With a few exceptions (notably the type strain DSM7^T^ CFS and BS27 CFS), all of the CFSs from this species showed some degree of inhibitory effect against all isolates of pathogenic *E. cecorum* tested. There was a greater predominance of positive inhibition results (vs. PC) among the *B. amyloliquefaciens* CFSs than was evident among the other *Bacillus* sp. CFSs, and the error bars accompanying the mean inhibition values produced by the *B. amyloliquefaciens* CFSs were markedly smaller (Figure 1). Comparison of the mean pathogen inhibition rates (%) by the *Bacillus* CFSs using ANOVA and Tukey's HSD confirmed that there were differences among *Bacillus* strains in their abi-lity to inhibit *E. cecorum* (*P* < 0.05) (Figure 1). A total of 8 CFSs, all of which were from commercial pro-biotic strains of *B. amyloliquefaciens*, exhibited average pathogen inhibition rates of >70% and small standard errors compared to the other strains tested, indicating a more consistent effect across pathogen strains. Five of these (CFSs 3AP4, 2084, ABP278, #1/1, and #10B/1) produced higher average inhibition rates (>75%) than 2 out of 3 *B. licheniformis* CFSs (#12/1 and #10/4, −2.5 and −8.39% vs. PC, respectively) and 1 out of 2 *B. subtilis* CFSs (#11/1, 7.3% vs. PC) (*P* < 0.05) (Figure 1). Commercial strain 3AP4 exhibited the highest mean percentage inhibition vs. PC and the lowest degree of variation across pathogen strains (85.0% ± 7.9). The lowest inhibition was produced by the CFS from the *B. amyloliquefaciens* type strain (DSM7^T^) which appeared to actively support growth of the pathogen (vs. PC) (mean % inhibition −39.3 ± 34.1).

**Figure 2.**
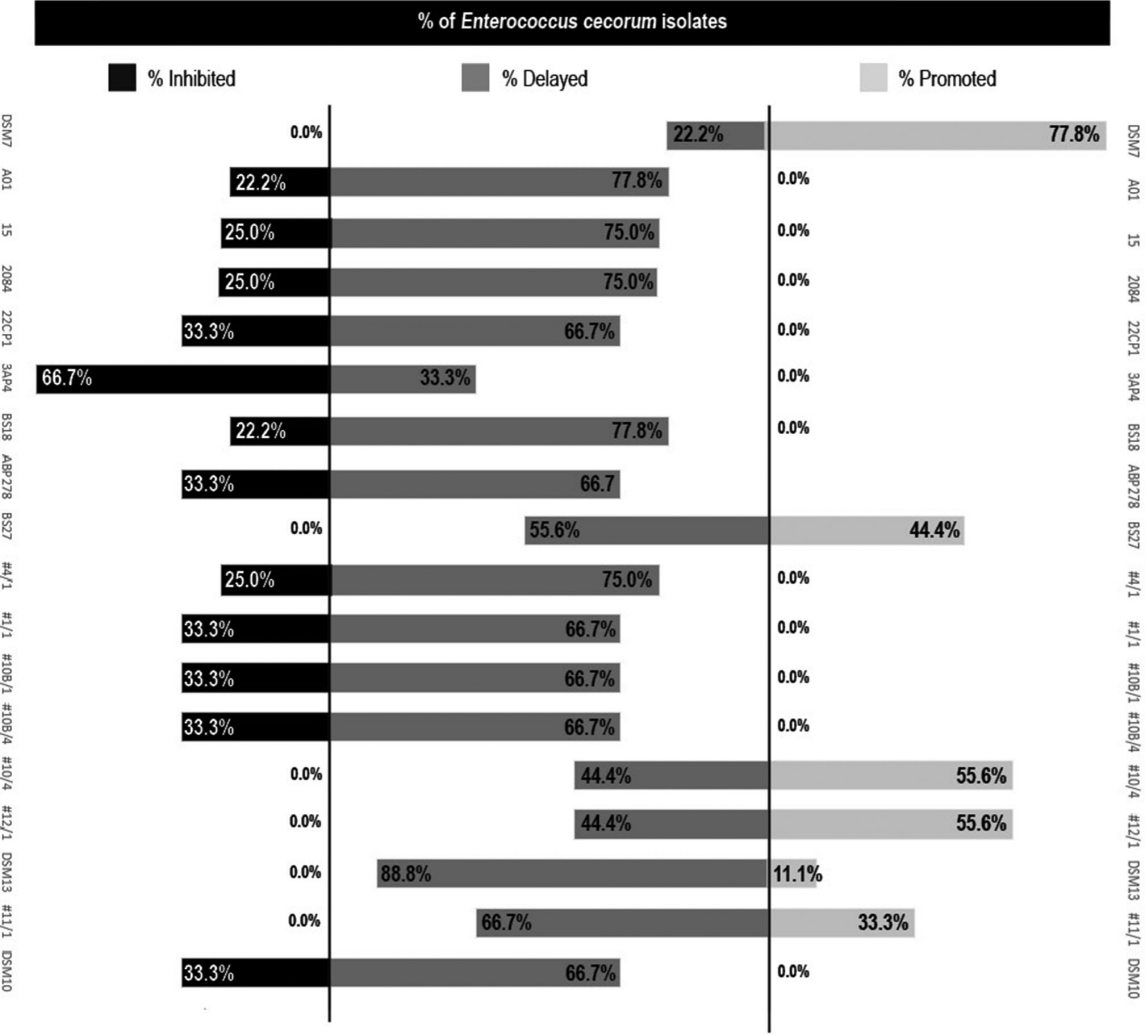
Percentage of pathogenic *E. cecorum* isolates inhibited, delayed, or promoted by cell-free supernatants of probiotic *Bacillus* spp.

The wider comparative ability of the different *Bacillus* CFSs to inhibit, delay or promote the growth of all 9 pathogenic *E. cecorum* isolates is shown in Figure 2. The most consistent inhibitory effect across *E. cecorum* isolates was produced by the CFS from *Bacillus* strain 3AP4, which inhibited the growth of 6 of the pathogenic strains for the entire 15 h experimental period, and delayed growth in the remaining 3 isolates by an average of 6.53 h ± 2.1. This means that, in the least efficacious scenario, CFS 3AP4 delayed the growth of the pathogen by at least 4 h. In contrast, *B. amyloliquefaciens* CFSs DSM7^T^, BS27, #10/4, #12/1, *B. licheniformis* CFSs DSM13 and #11/1 delayed the growth of 22.2 to 88.8% of the pathogenic isolates but did not fully inhibit any of them. Meanwhile, certain *Bacillus* CFSs promoted the growth of one or more of the *E. cecorum* isolates (CFSs obtained from the type strain DSM7^T^ and DSM13^T^; the internal negative control BS27, and 3 commercial probiotic strains #10/4, #12/1, and #11/1) (Figure 2).

## DISCUSSION

Competitive exclusion is a probiotic mode of action that can occur via several different mechanisms, one of which is the production of secondary metabolites or other antimicrobial substances that inhibit the growth of pathogens. Growth inhibitory and/or bacteriocidal effects against certain poultry pathogens in vitro have previously been described for a number of probiotic bacteria, including strains of *Bacillus* spp. (Svetoch et al., 2005; Teo and Tan, 2005; Latorre et al., 2016; Poormontaseri et al., 2017). The present study sought to extend the current knowledge by testing whether substances within the CFSs of commercially available probiotic and type strains of *Bacillus* spp. could inhibit the activity of clinical isolates of *E. cecorum* - a commensal bacteria present in healthy birds which is emerging as an opportunistic pathogen capable of causing significant clinical disease outbreaks.

The results revealed that CFSs from some of the *Bacillus* strains tested were effective at inhibiting the growth of clinical *E. cecorum* isolates in vitro, but this was not the case for all strains. Effects were highly *Bacillus*-strain dependent, both as to whether inhibitory activity was evident as well as the degree of effect observed. This varied from total (100%) inhibition for the duration of the experimental period (15 h), to delayed growth of the pathogen and partial inhibition, to an apparent promotion of pathogen growth of up to 100% compared with the PC (in which no *Bacillus* CFS was present). It is worth noting that three of the tested commercial strains that were highly effective at inhibiting pathogenic *E. cecorum* (CFSs 15AP4, BS8, and 2084) are used in combination in the commercial product DuPont™ Enviva^®^ PRO and as a combination should be more consistent than other single strain-based commercial products. The results here demonstrated that CFSs 15AP4, BS8, and 2084 have a diverse and a complementary coverage of inhibition.

The study's findings have highlighted significant strain specificity among probiotic *Bacillus* spp. in their in vitro effects on pathogenic microorganisms, namely pathogenic isolates of *E. cecorum*. This has not been reported previously and contrasts with recent concepts put forward by Sanders et al. (2018) that human clinical benefits of probiotics derived from *Bifidobacterium* and *Lactobacillus* genera are likely to derive from shared mechanisms at a sub-species, species, or genus level, rather than at strain level. Our findings suggest that, for *Bacillus*-based probiotics applied to poultry production, antimicrobial effects cannot be attributed at the species level. Nevertheless, the results also suggest that some of the existing proprietary *Bacillus* strains that are effective against major poultry pathogens may also be effective at controlling pathogenic *E. cecorum* growth and preventing gut colonization in vivo, whilst other strains may actively enhance growth of the pathogen. Thus, there may be potential for widening the application of the effective proprietary strains in poultry production. Under the in vitro conditions used here, CFSs from *B. amyloliquefaciens* proprietary strains 3AP4, 2084, ABP278, #1/1, and #10B/1 exhibited the most potent and consistent inhibitory effects against the *E. cecorum* isolates (>75% inhibition over the 15 h experimental period, on average, vs. the PC), whilst those from *B. licheniformis* strains #10/4 and #12/1, and *B. subtilis* strain #11/1 exhibited the lowest inhibitory effect (<10% inhibition, on average, vs. PC).

The *Bacillus* genus is known to be an extremely diverse taxonomic group, exhibiting huge genetic, phenotypic and functional variation, and differences between species and strains in relation to a range of functional properties and modes of probiotic action have been reported previously in the literature. Nevertheless, the apparent growth promotion effect of the CFSs from certain strains (BS27, #10/4, #12/1, #11/1) on the growth of some, but not all, of the *E. cecorum* isolates was unanticipated, and suggests that the antimicrobial activity of substances present in the CFSs was specific for particular *Bacillus* strain—*E. cecorum* isolate combinations. The CFS from the *B. amyloliquefaciens* type strain DSM7^T^, appeared to promote the growth of all the tested *E. cecorum* isolates under the study conditions (above the level seen in the PC), suggesting that substances present in the secretions of that particular strain were beneficial to *E. cecorum* growth. It is worth noting that type strains are not established based on any specific probiotic or pathogen-inhibitory activity per se, but rather as the definitive points of reference for a species (Lapage et al., 1990). Thus, it may be the case that the type strain DSM7^T^ exhibits quite different functional properties towards pathogens such as *E. cecorum* compared with strains that have been selected based on their probiotic properties. In fact, *E. cecorum* pathogen inhibitory activity varied markedly across the 3 type strains (DSM7^T^, DSM13^T^, and DSM10^T^) and was generally inconsistent with the observed activity of the proprietary probiotic strains. This would seem to indicate that the *Bacillus* type strains for *B. amyloliquefaciens*, *B. licheniformis*, and *B. subtilis* do not serve as reliable reference points for establishing the *E. cecorum* inhibitory activities of probiotic strains of these species.

The study design did not allow the identification of the inhibitory substances present in the CFSs tested, or their potency; the observed growth inhibitory effects could have been bacteriocidal or bacteriostatic in nature, or a combination of both. Five of the *Bacillus* strains (BS8, 15AP4, 2084, #1/1, and #10B/4) were effective at totally inhibiting growth of individual *E. cecorum* isolates for the duration of the experimental period (15 h), which would seem to suggest a bacteriocidal effect. Whilst for other *Bacillus* strain-pathogen isolate combinations growth was more moderately suppressed and, whilst delayed, appeared to otherwise track that seen in the control treatment (for example *Bacillus* strain CFS 22CP1 and CFS 15AP4 against *E. cecorum* isolate D4211). This is more suggestive of a bacteriostatic effect, in which reproduction is somewhat inhibited but the bacteria are not necessarily killed. Effects of some of the tested *Bacillus* strains were also more consistent (across *E. cecorum* isolates) than others, which may be a reflection of the concentration of antimicrobial substances released in the supernatant and/or to the variety of substances present. The pHs of all the CFSs were found to be broadly similar (between 6.4 and 6.8, data not shown), which may be reflective of their pH optima, this range being notably similar to the pH range found in the poultry small intestine (pH range 5.5 to 6.6; Shafey and McDonal, 1991), where effects would be manifested in vivo. Further studies are needed to isolate the active compound(s) and determine their precise mode(s) of action.

The inhabiting microbial community, physiological, and environmental conditions of the broiler small intestine are much more complex and variable than was represented by the controlled in vitro conditions of the study. Whether and to what degree the observed effects would be replicated in vivo, and what impact this might have on the ability of pathogenic *E. cecorum* to colonize the gut lining and cause clinical ES disease, remains to be determined. However, it seems likely that those *Bacillus* strains whose CFSs did not exhibit antimicrobial activity under study conditions (that excluded any competition effects) might be even less likely to do so in the more challenging environment of the gastrointestinal tract. The different pathogen growth kinetic responses to CFS indicated that where there was a delay, this frequently exceeded or approached the upper end of the 4 to 8 h average total tract retention time of the chicken (Svihus, 2010). This suggests that, if replicated in vivo, inhibitory substances contained in the extracellular secretions of the implicated strains may be effective in reducing opportunities for *E. cecorum* to colonize the gut lining of broilers. The potential transfer of ES disease from poultry to humans is a further factor to consider because *E. cecorum* is a zoonotic organism and in rare cases can cause human infections, presumably through animal–human transfer (Stubljar and Skvarc, 2015; Delaunay et al., 2015).

There is some evidence from existing studies of ES pathogenesis and of probiotic effects in poultry that suggest a second plausible mechanism of effect in vivo. The leakage of bacteria across the intestinal epithelial barrier and into the blood circulation is thought to be a key step in the pathogenesis of ES and is the route through which the bacteria gain access to bone sites for infection (Wideman, 2016). The integrity of this barrier is maintained by the activities of tight junction (TJ) complexes sited between adjacent epithelial cells, and a range of stress factors have been shown to be capable of compromising TJ activity, leading to a “leaky gut” in which there is increased opportunity for pathogens to cross the gut-epithelial barrier (Saunders et al., 1994; Quinteiro-Filho et al., 2010; Ulluwishewa et al., 2011). The ability of the commercial probiotic product Enviva^®^ PRO (which is a 1:1:1 combination of tested strains 15AP4, BS8, and 2084) to strengthen the gut barrier of the ileum and caecum in broilers and laying hens challenged with coccidia, *Campylobacter*, or *E. coli*, has already been demonstrated in vivo (Murugesan, 2013). Increased microbial challenge is one of the factors that can reduce TJ integrity, leading to the translocation of bacteria across the epithelium of the small intestine (Murugesan et al., 2014), and evidence suggests that certain *Bacillus*-based probiotics can enhance intestinal barrier integrity, prevent bacterial translocation in vitro and in vivo (Ulluwishewa et al., 2011; Pastorelli et al., 2013; Murugesan et al., 2014; Gadde et al., 2017) and support the immune system, as has been demonstrated in broilers for strains 15AP4, BS8, and 2084 (Amerah et al., 2013). No information about such effects in the presence of challenge with pathogenic strains of *E. cecorum* is currently available, but a dual-benefit hypothesis might be envisaged whereby strains of probiotic *Bacillus* could both enhance intestinal barrier activity at the same time as having a direct bacteriocidal or bacteriostatic effect on *E. cecorum* in the poultry small intestine.

In conclusion, the present study represents the first report of inhibitory activities of proprietary poultry *Bacillus* strains against pathogenic isolates of *E. cecorum* in vitro, but effects are highly strain dependent and vary significantly among different pathogenic isolates. This warrants the interest of a multi-strains probiotic product, especially because it is not *E. cecorum* specific and the variation in coverage is also true for other poultry pathogens (*E. coli*). Further work is required to establish whether these effects are also evident in vivo in broiler production conditions, as well as to isolate and characterize the specific inhibitory substances responsible for the observed effects.

## SUPPLEMENTARY DATA

**Supplementary material S1.** Examples of *E. cecorum* growth kinetics seen during exposure to different probiotic *Bacillus* strain cell-free supernatants.

**Supplementary material S2.** Percentage growth inhibition^1^ of pathogenic *E. cecorum* isolates by 18 *Bacillus* cell-free supernatants, measured at a time-point equivalent to that which produced an optical density of 0.4 in the positive control (obtained from duplicate).^1^ Defined as the percentage reduction in pathogen isolate growth in the experimental sample compared with that in the respective PC sample (containing pathogen in BHI medium but no *Bacillus* CFS), with growth being measured as biomass (absorbance OD), not as CFU, and being determined at the time-point equivalent to that producing an OD of 0.4 in the PC. Thus, 90% inhibition would mean a 90% reduction in growth (biomass). Note: values of greater than 100% occurred where the measured OD (nm) at time_x_ is less than the OD at time_0_. This may happen, for example, where lysis occurs in the pathogen+CFS suspension.

Supplemental File

## References

[bib1] Ahmed A. T., Islam Md. M., Mun H.-S., Sim H.-J., Kim Y.-J. 2014 Effects of *Bacillus amyloliquefaciens* as a probiotic strain on growth performance, cecal microflora, and fecal noxious gas emissions of broiler chickens. Poult. Sci. 93:1963–1971. 24902704 10.3382/ps.2013-03718

[bib2] Amerah A. M., Quiles A., Medel P., Sanchez J., Lehtinen M. J., Gracia M. I. 2013 Effect of pelleting temperature and probiotic supplementation on growth performance and immune function of broilers fed maize/soy-based diets. Anim. Feed Sci. Technol. 180:55–63.

[bib3] Aitchison H., Poolman P., Coetzer M., Griffiths C., Jacobs J., Meyer M., Bisschop S. 2014 Enterococcal-related vertebral osteoarthritis in South African broiler breeders: A case report. J. S. Afr. Vet. Assoc. 85:1077–1082. 25686375 10.4102/jsava.v85i1.1077

[bib5] Borst L. B., Suyemoto M. M., Scholl E. H., Fuller F. J., Barnes H. J. 2015 Comparative genomic analysis identifies divergent genomic features of pathogenic Enterococcus cecorum including a type IC CRISPR-Cas system, a capsule locus, an epa-like locus, and putative host tissue binding proteins. PLoS One. 10:e0121294. 25860249 10.1371/journal.pone.0121294 PMC4393107

[bib6] Borst L. B., Suyemoto M. M., Sarsour A. H., Harris M. C., Martin M. P., Strickand J. D., Oviedo E. O., Barnes H. J. 2017 Pathogenesis of Enterococcal Spondylitis caused by Enterococcus cecorum in broiler chickens. Vet. Pathol. 54:61–73. 27511310 10.1177/0300985816658098

[bib7] Cutting S. M. 2011 Bacillus probiotics. Food Microbiol. 28:214–220. 21315976 10.1016/j.fm.2010.03.007

[bib8] DeHerdt P., Defoort J., van Steelan H., Swam L., Tanghe S., Van Goethem M., Vanrobaeys M. 2008 *Enterococcus cecorum* osteomyelitis and arthritis in broiler chickens. Vlaams Diergeneeskundig Tijdschrift. 78:44–48.

[bib9] Delaunay E., Abat C., Rolain J.-M. 2015 *Enterococcus cecorum* human infection, France. New Microbes New Infect 7:50–51. 26199733 10.1016/j.nmni.2015.06.004 PMC4506978

[bib10] Devriese L. A., Dutta G. N., Farrow J. A. E., Vandekerckhove A., Philips B. A., 1983 *Streptococcus cecorum*, a new species isolated from chickens. Int. J. Syst. Bacteriol. 33:772–776.

[bib11] Devriese L. A., Ceyssens K., Haesebrouck F. 1991 Characteristics of *Enterococcus cecorum* strains from the intestines of different animal species. Lett. Appl. Microbiol. 12:137–139.

[bib12] Devriese L. A., Hommez J., Wijfels R., Haesebrouck F. 1991 Composition of the enterococcal and streptococcal intestinal flora of poultry. J. Appl. Microbiol. 71:46–50. 1910033

[bib13] Devriese L. A., Colque J. I. C., Deherdt P., Haesebrouck F. 1992 Identification and composition of the tonsillar and anal enterococcal and streptococcal flora of dogs and cats. J. Appl. Bacteriol. 73:421–425. 1447058 10.1111/j.1365-2672.1992.tb04998.x

[bib14] Devriese L. A. K. Cauwerts, Hermans K., Wood A. M. 2002 *Enterococcus cecorum* septicemia as a cause of bone and joint lesions resulting in lameness in broiler chickens. Vlaams Diergeneeskundig Tijdschrift. 71:219–221.

[bib15] Dolka B., Chrobak-Chmiel D., Makrai L., Szeleszczuk P. 2016 Phenotypic and genotypic characterization of *Enterococcus cecorum* strains associated with infections in poultry. BMC Vet. Res. 12:129. 27350248 10.1186/s12917-016-0761-1 PMC4924287

[bib16] Dolka B., Chrobak-Chmiel D., Czopowicz M., Szeleszczuk P. 2017 Characterization of pathogenic *Enterococcus cecorum* from different poultry groups: Broiler chickens, layers, turkeys, and waterfowl. PLoS One. 12:e0185199. 28934313 10.1371/journal.pone.0185199 PMC5608366

[bib17] FAO 2016 Probiotics in animal nutrition – Production, impact and regulation by Y. S. Bajagai, A. V. Klieve, P. J. Dart and Wayne L. Bryden. Makkar Harinder P. S., ed. FAO Animal Production and Health Paper No. 179, Rome.

[bib18] Fritts C. A., Kersey J. H., Motl M. A., Kroger E. C., Yan F., Si. Q. Jiang J., Campos M. M., Waldroup A. L., Waldroup P. W. 2000 *Bacillus subtilis* C-3102 (Calsporin) improves live performance and microbiological status of broiler chickens1. J. Appl. Poult. Res. 9:149–155.

[bib19] Gadde U., Oh S. T., S. Y. S. Lee, Davis E., Zimmerman N., Rehberger T. 2017 The effects of direct-fed microbial supplementation, as an alternative to antibiotics, on growth performance, intestinal immune status, and epithelial barrier gene expression in broiler chickens. Probiotics & Antimicro. Prot. 9:397–405. 10.1007/s12602-017-9275-9 28421423

[bib19a] Gebert S., Kromm C., Rehberger T. 2007 Effect of a Bacillus-based direct-fed microbial on turkey poultry performance and changes within the gastrointestinal microflora. Poult. Sci. 86(Suppl. 1):249.17234837

[bib20] Harada T., Kawahara R., Kanki M., Taguchi M., Kumeda Y. 2012 Isolation and characterization of vanA genotype vancomycin-resistant *Enterococcus cecorum* from retail poultry in Japan. Int. J. Food Microbiol. 153:372–377. 22192623 10.1016/j.ijfoodmicro.2011.11.029

[bib21] Jeong J. S., Kim I. H. 2014 Effect of *Bacillus subtilis* C-3102 spores as a probiotic feed supplement on growth performance, noxious gas emission, and intestinal microflora in broilers. Poult. Sci. 93:3097–3103. 25260523 10.3382/ps.2014-04086

[bib22] Jung A., Rautenshlein S. 2014 Comprehensive report of an Enterococcus cecorum infection in a broiler flock in Northern Germany. BMC Vet. Res. 10:311. 25539747 10.1186/s12917-014-0311-7 PMC4297365

[bib23] Lapage S. P., Sneath P H. A., Lessel E. F., Skerman V. B. D., Seeliger H. P. R., Clark W. A., (eds.). 1990 Revision International Code of Nomenclature of Bacteria, ASM Press, Washington, DC. 21089234

[bib24] Latorre J. D., Hernandez-Velasco X., Wolfenden R. E., Vicente J. L., Wolfenden A. D., Menconi A., Bielke L. R., Hargis B. M., Tellez G. 2016 Evaluation and selection of *Bacillus* species based on enzyme production, antimicrobial activity, and biofilm synthesis as direct-fed microbial candidates for poultry. Front. Vet. Sci. 3:95. 27812526 10.3389/fvets.2016.00095 PMC5071321

[bib25] Lee K., Lillehoj H. S., Siragusa G. R. 2010 Direct-fed microbials and their impact on the intestinal microflora and immune system of chickens. J. Poult. Sci. 47:106–114.

[bib26] Lei X., Piao X., Ru Y., Zhang H., Peron A., Zhang H. 2015 Effect of *Bacillus amyloliquefaciens*-based direct-fed microbial on performance, nutrient utilization, intestinal morphology and cecal microflora in broiler chickens. Asian Australas. J. Anim. Sci. 28:239–246. 25557820 10.5713/ajas.14.0330 PMC4283169

[bib27] Martin L. T., Martin M. P., Barnes H. J. 2011 Experimental reproduction of enterococcal spondylitis in male broiler breeder chickens. Avian Dis. 55:273–278. 21793445 10.1637/9614-121410-Reg.1

[bib28] Murugesan G. R. 2013 Characterization of the effects of intestinal physiology modified by exogenous enzymes and direct-fed microbials on intestinal integrity, energy metabolism, body composition and performance of laying hen and broiler chicks. PhD Thesis No. 13175 Iowa State University, Ames, Iowa.

[bib29] Murugesan G. R., Gabler N. K., Persia M. E. 2014 Effects of direct-fed microbial supplementation on broiler performance, intestinal nutrient transport and integrity under experimental conditions with increased microbial challenge. Br. Poult. Sci. 55:89–97. 24219515 10.1080/00071668.2013.865834

[bib30] Pastorelli L., De Salvo C., Mercado J. R., Vecchi M., Pizarro T. T. 2013 Central role of the gut epithelial barrier in the pathogenesis of chronic intestinal inflammation: lessons learned from animal models and human genetics. Frontiers in Science (Front. Immunol.) 4:280. 10.3389/fimmu.2013.00280 PMC3775315 24062746

[bib31] Poormontaseri M., Hosseinzadeh S., Shekarforoush S. S., Kalantari T. 2017 The effects of probiotic *Bacillus subtilis* on the cytotoxicity of *Clostridium perfringens* type a in Caco-2 cell culture. BMC Microbiol. 17:150. 28676033 10.1186/s12866-017-1051-1 PMC5496268

[bib32] Quinteiro-Filho W. M., Ribeiro A., Ferraz-de-Paula V., Pinheiro M. L., Sakai M., Sá L. R., Ferrieira J. P., Palermo-Neto J. 2010 Heat stress impairs performance parameters, induces intestinal injury, and decreases macrophage activity in broiler chickens. Poult. Sci. 89:1905–1914. 20709975 10.3382/ps.2010-00812

[bib33] Sanders M. E., Benson A., Lebeer S., Merenstein D. J., Klaenhammer T. R. 2018 Shared mechanisms among probiotic taxa: Implications for general probiotic claims. Curr. Opin. Biotechnol. 49:207–216. 29128720 10.1016/j.copbio.2017.09.007

[bib34] Saunders P. R. U., Kosecka U., McKay D. M., Perdue M. H. 1994 Acute stressors stimulate ion secretion and increase epithelial permeability in rat intestine. Am. J. Physiol. 267:G794–G799. 7977741 10.1152/ajpgi.1994.267.5.G794

[bib35] Shafey T. M., McDonal M. W. 1991 The effects of dietary calcium, phosphorus, and protein on the performance and nutrient utilization of broiler chickens. Poult. Sci. 70:548–553. 2047348 10.3382/ps.0700548

[bib36] Stubljar D., Skvarc M. 2015 Enterococcus cecorum infection in two critically ill children and in two adult septic patients. Slov. Vet. Res. 52:39–44.

[bib37] Svetoch E. A., Stern N. J., Eruslanov B. V., Kovalev Y. N., Volodina L. I., Perelygin V. V., Mitsevich E. V., Mitsevich I. P., Pokhilenko V. D., Borzenkov V. N., Levchuk V. P., Svetoch O. E., Kudriavtseva T. Y. 2005 Isolation of *Bacillus circulans* and *Paenibacillus polymyxa* strains inhibitory to Campylobacter jejuni and characterization of associated bacteriocins. J. Food Prot. 68:11–17. 15690798 10.4315/0362-028x-68.1.11

[bib4] Svihus B. 2010 Effect of digestive tract conditions, feed processing and ingredients on response to NSP enzymes. Pages 129–159 in Enzymes in Farm Animal Nutrition Bedford M. R., Partridge G. G., eds. CABI Wallingford, UK.

[bib38] Teo A. Y., Tan H. M. 2005 Inhibition of *Clostridium perfringens* by a novel strain of Bacillus subtilis isolated from the gastrointestinal tracts of healthy chickens. Appl. Environ. Microbiol. 71:4185–4190. 16085801 10.1128/AEM.71.8.4185-4190.2005 PMC1183296

[bib39] Ulluwishewa D., Anderson R. C., Mcnabb W. C., Moughan P. J., Wells J. M., Roy N. C. 2011 Regulation of tight junction permeability by intestinal bacteria and dietary components. J. Nutr. 141:769–776. 21430248 10.3945/jn.110.135657

[bib40] Wideman R. F. 2016 Bacterial chondronecrosis with osteomyelitis and lameness in broilers: A review. Poult. Sci. 95:325–344. 26527707 10.3382/ps/pev320

[bib41] Wood A. M., MacKenzie N. C., Brown L., Devriese L. A., Baele M. 2002 Isolation of *Enterococcus cecorum* from bone lesions in broiler chickens. Vet. Rec. 150:27. 11822370

[bib42] Wu B. Q., Zhang T., Guo L. Q., Lin J. F. 2011 Effects of Bacillus subtilis KD1 on broiler intestinal flora. Poult. Sci. 90:2493–2499. 22010234 10.3382/ps.2011-01529

