# Supplementary material for: In vitro inhibition of avian pathogenic Enterococcus cecorum isolates by probiotic Bacillus strains
Source: Poult Sci. 2019 Jan 18;98(6):2338–46. doi: 10.3382/ps/pey593 (PMC6527510; doi:10.3382/ps/pey593)
Supplement: Supplemental File [file pey593_supplement_file.docx]

**Supplementary material**

**Supplementary material S1.** Examples of *E. cecorum* growth kinetics seen during exposure to different probiotic *Bacillus* strain cell-free supernatants.

**a)**

**b)**

**c)**

**d)**

**e)**

**Supplementary material S2.** Percentage growth inhibition^1^ of pathogenic *E. cecorum* isolates by 18 *Bacillus* cell-free supernatants, measured

at a time-point equivalent to that which produced an optical density of 0.4 in the positive control (obtained from duplicate).

^1^Defined as the percentage reduction in pathogen isolate growth in the experimental sample compared with that in the respective PC sample (containing pathogen in BHI medium but no *Bacillus* CFS), with growth being measured as biomass (absorbance (OD)), not as CFU, and being determined at the time-point equivalent to that producing an OD of 0.4 in the PC. Thus, 90% inhibition would mean a 90% reduction in growth (biomass).

Note: values of greater than 100% occurred where the measured OD (nm) at time_x_ is less than the OD at time_0_. This may happen, for example, where lysis occurs in the pathogen+CFS suspension.
